# Cardiovascular adaptation to simulated microgravity and countermeasure efficacy assessed by ballistocardiography and seismocardiography

**DOI:** 10.1038/s41598-020-74150-5

**Published:** 2020-10-19

**Authors:** Jeremy Rabineau, Amin Hossein, Federica Landreani, Benoit Haut, Edwin Mulder, Elena Luchitskaya, Jens Tank, Enrico G. Caiani, Philippe van de Borne, Pierre-François Migeotte

**Affiliations:** 1 LPHYS, Université Libre de Bruxelles, Brussels, Belgium; 2 TIPs, Université Libre de Bruxelles, Brussels, Belgium; 3 Electronic, Information and Biomedical Engineering Department, Politecnico Di Milano, Milan, Italy; 4 Institute of Aerospace Medicine, German Aerospace Center (DLR), Cologne, Germany; 5 Institute of Biomedical Problems of the Russian Academy of Sciences, Moscow, Russian Federation; 6 Department of Cardiology, Erasme Hospital, Université Libre de Bruxelles, Brussels, Belgium

**Keywords:** Cardiology, Blood flow

## Abstract

Head-down bed rest (HDBR) reproduces the cardiovascular effects of microgravity. We tested the hypothesis that regular high-intensity physical exercise (JUMP) could prevent this cardiovascular deconditioning, which could be detected using seismocardiography (SCG) and ballistocardiography (BCG). 23 healthy males were exposed to 60-day HDBR: 12 in a physical exercise group (JUMP), the others in a control group (CTRL). SCG and BCG were measured during supine controlled breathing protocols. From the linear and rotational SCG/BCG signals, the integral of kinetic energy ($iK$) was computed on each dimension over the cardiac cycle. At the end of HDBR, BCG rotational $iK$ and SCG transversal $iK$ decreased similarly for all participants (− 40% and − 44%, respectively, *p* < 0.05), and so did orthostatic tolerance (− 58%, *p* < 0.01). Resting heart rate decreased in JUMP (− 10%, *p* < 0.01), but not in CTRL. BCG linear $iK$ decreased in CTRL (− 50%, *p* < 0.05), but not in JUMP. The changes in the systolic component of BCG linear *iK* were correlated to those in stroke volume and V_O2_ max (R = 0.44 and 0.47, respectively, *p* < 0.05). JUMP was less affected by cardiovascular deconditioning, which could be detected by BCG in agreement with standard markers of the cardiovascular condition. This shows the potential of BCG to easily monitor cardiac deconditioning.

## Introduction

Prolonged exposure to weightlessness is known to cause a cascade of cardiovascular adaptations starting with a head-ward shift of blood volume^1^. These adaptations are well reproduced on Earth when individuals are exposed to head-down (6°) tilt bed rest (HDBR)^2^, which induces a similar blood shift and a lack of arterial baroreceptor input, as seen during space flight^2^. Previous investigations have shown that exposure to real or simulated weightlessness causes severe deconditioning of the cardiovascular system, as manifested by a decrease in plasma volume, red cell mass^1^, and stroke volume (SV), an apparent reduction in left ventricular mass^3,4^, as well as a decrease in maximal aerobic capacity^5^. At the same time, cardiac compliance increases^6^, carotid arteries stiffen^7^, intima-media thickness increases^8^, while some vascular changes occur also in the lower limbs^2,9^. Numerous additional observations have been reported regarding modifications of aortic^10^, carotid^11^, and cardiopulmonary^12^ baroreflexes. Collectively, these changes substantially compromise orthostatic tolerance following exposure to a weightlessness environment^13^. However, appropriate cardiovascular countermeasures applied during (simulated) weightlessness can limit this deconditioning and these symptoms disappear already 3 to 5 days after return to normal ambulation^14^.

Unlike spaceflight conditions, experimental HDBR studies are extremely well controlled in terms of physical activity, dietary intake, sleep duration, etc. Furthermore, HDBR subjects are more easily accessible to perform extensive testing, making HDBR an ideal model for observing progressive cardiovascular adaptations during prolonged weightlessness, as well as testing and validating new techniques and devices against gold standard methods.

In space, it is very often impossible to use gold standard techniques to assess cardiovascular health and the inotropic state of the heart. Hardware allowing for cardiovascular magnetic resonance (CMR) imaging is not available, while the ultrasound system currently available onboard the international space station (ISS) is underused because of the extensive training required for the astronauts to be able to perform cardiac measurements. Remotely guided tele-echocardiography has been successfully tested together with e-training methods, even if poor data obtained from some echocardiographic windows prevented a complete analysis^15^. The use of a tele-operated ultrasound system with motorized probes has shown to help reducing the training constraints, but still requires the downlink of real-time video and two-way audio with a short time-delay and a sufficient bandwidth^16^. This limitation will only be exacerbated in future exploration-class missions.

In this context, there would be an obvious benefit to have an easy-to-use portable device that would not require the help of any external operator to assess cardiac mechanical health. So far, the portable cardiac monitoring (PCM) tools focus mainly on chronotropy (e.g. ECG Holter monitors). However, for all the reasons mentioned up to this point, wearable or portable devices for assessment of inotropy would be very important in space.

The research presented in this paper was part of the European Space Agency (ESA) “Reactive jumps in a Sledge jump system as a countermeasure during Long-term bed rest” (RSL) HDBR study. We hypothesized that an easy-to-use PCM device that combines electrocardiography (ECG), seismocardiography (SCG), and six-dimension ballistocardiography (BCG), would be sensitive enough to monitor changes in cardiac inotropic state caused by HDBR, but also to determine the efficacy of an exercise-based countermeasure.

BCG represents a measurement of the global movements of the body in reaction to the cardiac ejection of blood into the vasculature, while SCG is a measurement of the precordial vibrations in response to the heartbeat^17^. BCG has been used already in the early days of human exposure to weightlessness, during parabolic^18^ and space flight^19^, in order to get a pure multi-dimensional signal without dampening of the body support. Several of these studies have shown the importance to measure BCG on three axes, rather than only in the head-to-foot direction, as was done so far with most of the terrestrial BCG devices^20,21^. Moreover, other studies have evidenced some changes in the spatial distribution of the BCG signal in weightlessness, compared to normal Earth conditions^22^. Despite being easy-to-use, non-invasive, and non-obtrusive techniques, thus far BCG and SCG have been tested only on a very small number of subjects in weightlessness, and results were not compared to any gold standard measurement of cardiac condition.

The RSL study offered the unique possibility to longitudinally assess BCG and SCG while the cardiovascular system was gradually deconditioning during 60 days of HDBR. Consequently, the main objective of this work was to evaluate the effect of HDBR-induced cardiovascular deconditioning on the BCG and SCG metrics, and to evaluate if these techniques would be sensitive enough to differentiate between control and countermeasure groups. This study also offered the opportunity to compare the evolution of BCG and SCG metrics to the ones of other markers of the cardiovascular health during HDBR. However, this should be seen as a secondary objective bringing only informative value, since the study was not initially designed for this purpose. If proven convincing, BCG and SCG would enable us to monitor in-orbit cardiac health in astronauts, where the use of echocardiography and CMR are either limited, or even absent.

## Methods

### Design of the study

The research presented in this paper, which was part of the ESA-RSL study, took place at the :envihab facility of the German Aerospace Center (DLR) in Cologne, Germany. This HDBR study consisted in a randomized controlled single-center, parallel-group study, conducted during two successive campaigns that started in August 2015 and in January 2016, respectively.

**Figure 1:**
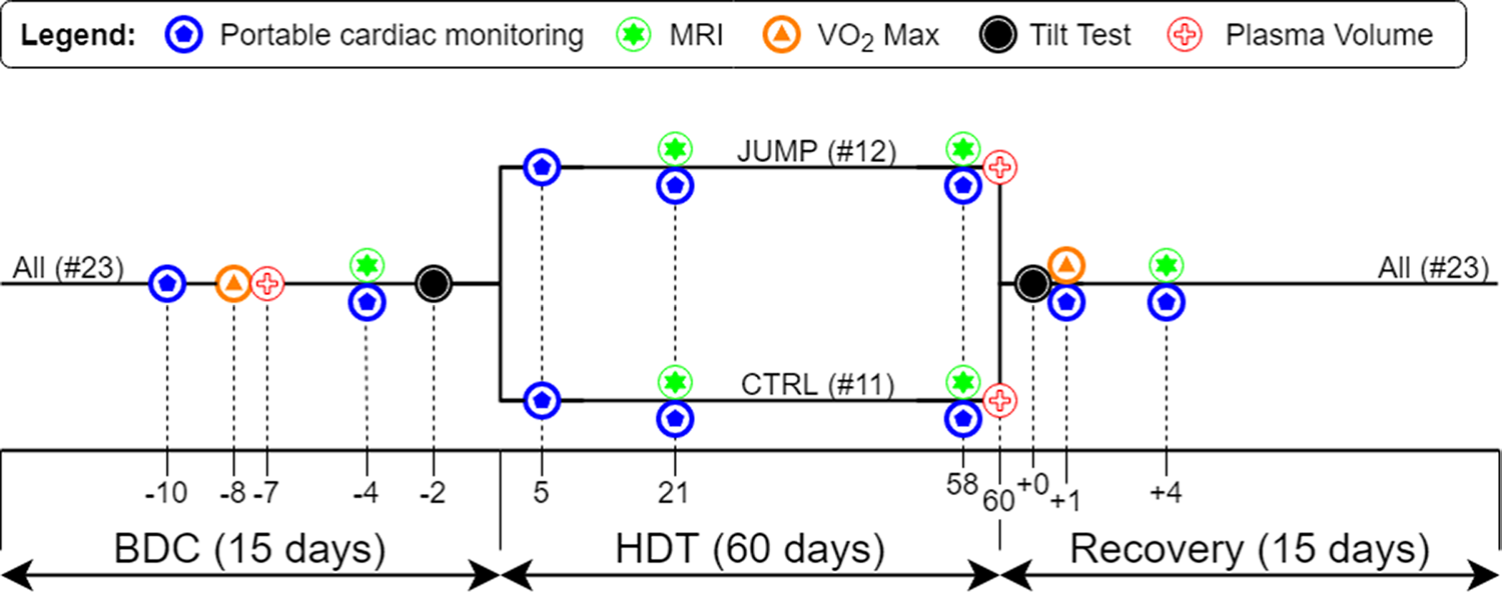
General overview of the ESA-RSL bed rest study. *BDC* baseline data collection, *HDT* head-down tilt.

During each campaign, the participants were distributed in pairs, having the same activities on the same days, except for the countermeasure protocol. Each campaign started with the participants spending 15 days of normal ambulation at :envihab for familiarization and baseline data collection (BDC-15 to BDC-1, see Fig. 1). At the end of this first period, in each pair of participants, one participant was randomly assigned to the countermeasure group (JUMP) and the other one to the control group (CTRL). Then, they all started a period of 60-day continuous 6° head-down tilt (HDT) bed rest (HDT1 to HDT60). Re-ambulation of the participants occurred after 60 days of HDT bed rest and corresponded to the beginning of a 15-day recovery period (R + 0 to R + 14), still performed at :envihab. During recovery, each subject participated in six 30-min sessions of personalized reconditioning to facilitate recovery of muscle strength, speed, coordination, balance, etc.

The acronym “HDBR” will be used to refer to the overall experiment, with its distinct three phases: BDC, HDT, and recovery. “HDT” will refer only to the head-down tilt position or to the period when the subjects were in this position.

During the HDT period, the JUMP group performed physical training five to six times per week (total of 48 training sessions during the 60 days of HDT). Physical training consisted of performing squats, heel raises, hops, and countermovement jumps against a resistive force, while lying in horizontal position on a sledge jump system (Novotec Medical GmbH, Pforzheim, Germany)^23^. On average, a training session comprised of 6 series of 12 jumps, with an overall duration of about 3 min of physical activity. Details regarding the organization of the ESA-RSL study as well as the design of the countermeasure performed by the JUMP group is given by Kramer et al.^23^.

The study protocol complied with the Declaration of Helsinki and was approved by the ethics committees of the Northern Rhine Medical Association as well as the German Federal Office for Radiation Protection. It was registered at the German Clinical Trial Registry (DRKS, registration number: DRKS00012946). Written informed consent was obtained from each participant before the beginning of the study.

### Recruitment and participants

The following inclusion criteria were used for the recruitment of the ESA-RSL study: male, between 20 and 45 years old, body mass index (BMI) between 20 and 26 kg/m^2^, non-smoker, no medication, no competitive athlete, and no history of bone fracture. Exclusion criteria were the following: chronic hypertension, diabetes, obesity, arthritis, hyperlipidaemia, hepatic disease, disorder of calcium bone metabolism, or heritable blood clotting disorders. In addition, the selection process included a psychological screening as well as a screening of bone mineral density of the proximal femur and the lumbar vertebra by dual energy X-ray absorptiometry (DXA).

A total of 24 healthy male subjects (29 ± 6 years old) were included. One subject had to be excluded from further participation during the BDC period for medical reasons non-related to the study, leading to a total number of 23 participants completing the HDBR study. One participant that was initially assigned to the JUMP group was reallocated to the CTRL group after three training sessions, because of a possible medial tibia stress syndrome. In addition, due to medical reasons non-related to the study, one CTRL and one JUMP subject were re-ambulated before the end of the HDT period (at HDT49 and HDT50, respectively). This caused their recovery schedule to start sooner, but they completed all the measurements planned both for the bed rest phase, as well as the recovery period. The demographic details of both study groups are given in Table 1.

**Table 1:** Baseline data of the two groups of participants of the ESA-RSL study. N represents the number of participants in each group; *BMI* Body Mass Index, *CC* cardiac cycle, *sys* systole, *dia* diastole. Values are given as median [Q1; Q3].

Metrics		BDC CTRL	BDC JUMP	Intergroup difference
N		10	12	–
Age (year)		28 [24; 34]	28 [25; 33]	*p* = 0.93
Weight (kg)		77 [68; 83]	80 [73; 82]	*p* = 0.89
Height (cm)		180 [176; 181]	182 [178; 186]	*p* = 0.63
BMI (kg/m^2^)		24.5 [22.3; 25.2]	23.4 [22.6; 24.9]	*p* = 0.63
Heart rate (bpm)		66 [52; 70]	67 [62; 76]	*p* = 0.23
${{{i}}{{K}}}_{{{L}}{{i}}{{n}}}^{{{B}}{{C}}{{G}}}$(µJ s)	*CC*	2.8 [2.0; 3.5]	1.8 [1.8; 2.3]	*p* = 0.02
	*sys*	1.6 [1.2; 2.0]	1.2 [1.0; 1.3]	*p* = 0.09
	*dia*	1.1 [0.7; 1.7]	0.7 [0.5; 1.1]	*p* = 0.43
${{{i}}{{K}}}_{{{R}}{{o}}{{t}}}^{{{B}}{{C}}{{G}}}$(µJ s)	*CC*	6.5 [4.6; 12.3]	10.7 [4.3; 13.5]	*p* = 0.70
	*sys*	4.7 [3.3; 9.0]	6.5 [3.2; 11.2]	*p* = 0.83
	*dia*	2.0 [1.5; 2.3]	1.9 [1.0; 2.9]	*p* = 0.83
${{{i}}{{K}}}_{{{z}}}^{{{S}}{{C}}{{G}}}$(µJ s)	*CC*	22.8 [11.4; 37.7]	23.0 [15.4; 28.2]	*p* = 0.49
	*sys*	15.2 [7.3; 22.8]	11.9 [8.2; 21.7]	*p* = 0.88
	*dia*	7.4 [5.7; 13.7]	8.0 [6.0; 9.7]	*p* = 0.88
Orthostatic tolerance (min)		22.4 [20.3; 24.7]	23.3 [22.2; 23.9]	*p* = 0.70
Stroke volume (ml)		110 [102; 130]	104 [99; 110]	*p* = 0.43
V_O2_ max (ml/kg/min)		51.1 [49.0; 52.3]	41.3 [34.5; 46.7]	*p* = 0.01
Plasma volume (l)		3.8 [3.3; 4.3]	3.5 [3.4; 4.1]	*p* = 0.63

### Portable cardiac monitoring

#### Material

**Figure 2:**
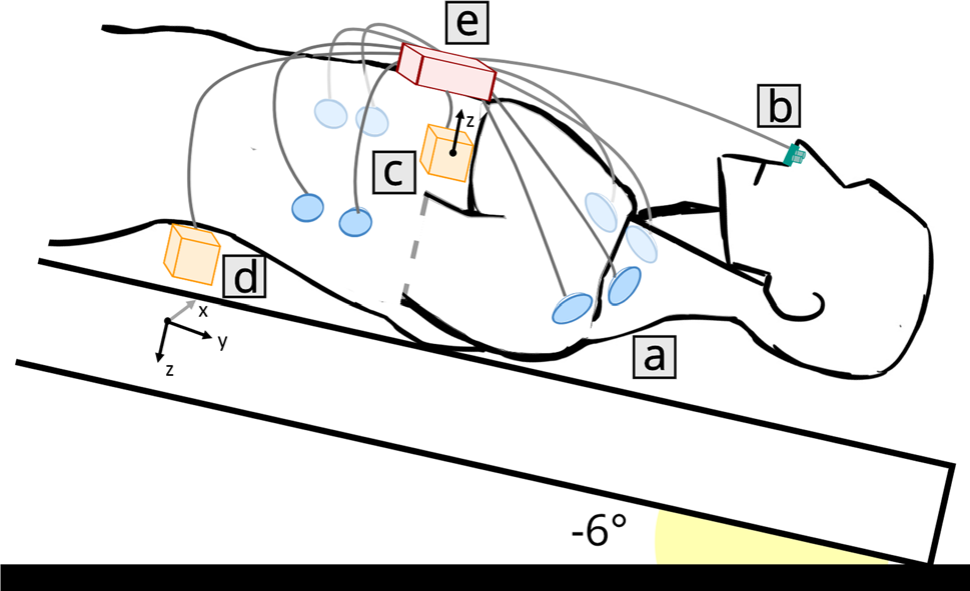
A schematic representation of the different elements of CARDIOVECTOR-1. (**a**) ECG/ICG electrodes; (**b**) PTG sensor (nasal thermistor); (**c**) SCG sensor at the cardiac apex (dorsoventral linear accelerations); (**d**) BCG sensor between the second and the third lumbar vertebrae (3-axis linear accelerations and 3-axis angular velocities); (**e**) Main unit (connection and amplification).

The PCM was performed using a modified version of CARDIOVECTOR-1 (Medical Computer Systems Ltd., Zelenograd, Russian Federation). This device has already been described by Baevsky and colleagues^22^ and used in space^24^. It allows the synchronous acquisition of: (a) electrocardiography (ECG) and impedance cardiography (ICG) in tetrapolar configuration; (b) plethysmography (PTG) using a nasal thermistor to evaluate breathing; (c) 1-axis dorsoventral seismocardiography (SCG, linear accelerations recorded at the cardiac apex); and (d) 6 degrees of freedom ballistocardiography (BCG, 3-axis linear accelerations and 3-axis angular velocities, recorded between the second and the third lumbar vertebrae, close to the participant’s center of mass). The sensors as well as the connection and amplification unit were attached to the body of the participants with plaster (see Fig. 2). The BCG sensor was secured with a Velcro belt. The amplification unit was connected to a computer that allowed the acquisition of the signals at a sampling frequency of 1,000 Hz for all channels. The standard nomenclature was used for the direction and the orientation of the BCG and SCG axes^17,25^.

#### Experimental protocol

After being instrumented in supine position (6° HDT during bed rest; horizontal during BDC and recovery periods), subjects were instructed to remain as still as possible without moving, talking, or falling asleep during the entire recording phase.

As the influence of breathing on accelerometric data, such as the BCG, is well known^21,26,27^, acquisition was standardized through an imposed and controlled breathing (ICB) protocol to reduce intra- and inter-subject variability, possibly due to differences in spontaneous breathing. This ICB protocol consisted of 4 sequences of 10 repetitions of a fixed length (4, 6, 8, and 10 s) breathing cycle (half for inspiration, half for expiration). Subjects were instructed and guided through the ICB protocol via a visual display indicating the breathing pattern to follow during the measurement.

After being familiarized with the equipment and the ICB protocol, measurements were obtained at BDC-4, HDT5, HDT21, HDT58, R + 1, and R + 4 (see Fig. 1). For one subject, the baseline measurement at BDC-4 was not acquired, so the familiarization acquisition (BDC-10) was used instead. The two subjects that were prematurely re-ambulated performed the final HDT measurement at HDT48 and HDT49, respectively, instead of HDT58.

#### Post-hoc signal processing

Beat to beat R waves were automatically detected from the raw ECG signal, checked visually, and corrected when needed. These R waves were used as fiducial points to delimitate the cardiac beats through the record. For each of the 4 phases of the ICB protocol (4-, 6-, 8-, and 10-s breathing cycles), an ensemble average for the ECG signal and for all BCG and SCG channels was computed. Because there were no differences between the various ICB breathing durations, only data from the 6-s breathing protocol, corresponding to a relaxed breathing pattern, are presented. Based on the ensemble averaged ECG signal, a systolic and a diastolic phase were defined: the systolic phase starts at the Q wave and finishes at the end of the T wave, while the diastolic phase corresponds to the rest of the heartbeat, from the end of the T wave until the next Q wave. The delimitation of the systolic/diastolic phases have both been checked visually on each ensemble averaged signal and corrected when needed.

From the ensemble averaged SCG and BCG signals, three instantaneous kinetic energies (K) transmitted to the sensors by the cardiac activity were computed based on each subject’s inertial parameters (body mass for the linear channels; matrix of inertia for the rotational channels, as estimated using a model based on the height and weight of the subject^28^): namely (a) the linear BCG kinetic energy (regrouping x, y, and z), (b) the rotational BCG kinetic energy (regrouping x, y, and z), and (c) the SCG kinetic energy along the linear z axis. These computations gave the following ensemble-averaged signals: ${K}_{Lin}^{BCG}$, ${K}_{Rot}^{BCG}$, ${K}_{z}^{SCG}$, respectively. Finally, corresponding $iK$ parameters, each equal to the integral of a kinetic energy on a given cardiac cycle interval (CCI), were computed:

1 $$iK~CCI={\int }_{CCI}K\left(t\right) . dt$$

where CCI, based on the ensemble-averaged ECG, can either be the whole cardiac cycle (CC), the systolic (sys), or the diastolic (dia) phase, as defined previously. Complementary information regarding the signal processing of multi-dimensional BCG and SCG signals are reported elsewhere^29^.

Of the planned 138 records (6 time points for 23 subjects), some technical issues led to 3 altered SCG signals (concerning 3 different subjects) and 9 missing measurements for the linear z axis of BCG (concerning 9 different subjects). To ensure that the time points would be comparable amongst all subjects, we decided to compute the linear BCG metrics on the 138 records using only the x and y axes. The recordings of one CTRL subject were excluded from analyses, because the computed metrics for this subject appeared as clear outliers.

### Standard tests

Each HDBR study organized by ESA includes a set of standard tests forming the so called “bedrest core data”. Among them are tests that are relevant to the cardiovascular state: maximal aerobic capacity, plasma volume, and orthostatic tolerance. The planning of these tests is presented in Fig. 1. Maximal aerobic capacity, plasma volume, and SV results for the ESA-RSL study have already been published by Kramer and coworkers^23,30^, as well as Caiani and coworkers^31^, and are discussed in regard to the portable monitoring results presented in this study.

#### Orthostatic tolerance

The orthostatic tolerance test was performed using an electrically driven tilt table equipped with a lower body negative pressure (LBNP) chamber, close to the start of bed rest (BDC-2), and at the very first day (R + 0) of re-ambulation (this test ended the HDT period, see Fig. 1). After 20 min rest in supine position, the orthostatic tolerance test started with the subject tilted to an 80° head-up position, that was maintained for 15 min, or until pre-syncope symptoms appeared, marking the end of the test. If none of these symptoms were observed, LBNP at − 10 mmHg was applied for 3 min, with additional increments of − 10 mmHg in 3 min stages until pre-syncope. During the entire procedure, the subject was discouraged from movement, muscle contractions, and talking. Vital signs were monitored via a 3-lead ECG (Datex Ohmeda, GEHealthcare, Helsinki, Finland) and oscillometric blood pressure every third minute (Datex Ohmeda, GEHealthcare, Helsinki, Finland). Blood pressure was also continuously recorded at the finger (Finometer, TNO, Amsterdam, the Netherlands) resulting in model flow estimates for beat-to-beat SV and cardiac output. In addition, an impedance cardiography technique (Biopac systems inc., Goleta, CA, USA) was used to record the parameters associated with cardiac output measurements. Orthostatic tolerance time was defined as the time elapsed from the beginning of the head-up tilt until the emergence of pre-syncope symptoms. Termination criteria were a sudden drop in heart rate (more than 15 bpm), a significant drop in blood pressure, significant cardiac arrhythmias, severe nausea, lightheadedness, or pain, breathing difficulty, loss of motor activity in any extremity, or subject noncompliance.

#### Stroke volume

SV was measured by MRI (Biograph mMR 3-T scanner, Siemens, Erlangen, Germany) once during the BDC phase (BDC-4), twice during the HDT phase (HDT21 and HDT58), and once during the recovery phase (R + 4, see Fig. 1). The subject was placed inside the MRI machine in supine 0° position. 2D PC-MRI images (30 frames per cardiac cycle, spatial resolution 1.4 × 1.4 × 5.0 mm^3^) were acquired during spontaneous breathing using a plane perpendicular to the centerline at a proximal site of the ascending aorta, with three-directional velocity encoding (x, y: 80 cm/s; z: 150 cm/s). SV was computed as the time integral of the flow rate in the systolic ejection period. More information concerning this protocol is given by Caiani and coworkers^31^.

#### Maximal aerobic capacity

A cycle ergometer (Lode, Groningen, the Netherlands) was used to measure the maximal oxygen capacity relative to the body weight (V_O2_ max) during the BDC phase (BDC-8) and shortly after return to normal ambulation (R + 1, see Fig. 1). The subject was asked to cycle at a constant cadence with increasing load, until exhaustion. O_2_ uptake and CO_2_ emission were monitored with the Innocor system (Innovision, Odense, Denmark) and heart rate was monitored with a 12-lead ECG (Padsy, Medset Medizintechnik, Germany). More information concerning this protocol is given by Kramer and coworkers^30^. Two subjects could not complete this test at R + 1.

#### Plasma volume

The Schmidt CO rebreathing technique^32^ was used to measure blood volume and composition on all the participants during the BDC phase (BDC-7) and on the last day of HDT (HDT60). Before starting the protocol, a 20-min resting period was conducted in horizontal supine position at BDC-7 and in HDT position at HDT60. Then, the participant was connected to a Krogh-spirometer (Student Spirometer, ZAK, Germany) and started the rebreathing procedure. More information concerning this protocol is given by Kramer and coworkers^23^.

### Statistical analysis

The values of the metrics acquired by PCM were assessed once before bed rest (BDC-4, considered as the baseline value), three times during HDT (HDT5, HDT21, and HDT58), and twice during recovery (R + 1, R + 4). SV was measured at BDC-4, HDT21, HDT58, and R + 4. Orthostatic tolerance, plasma volume and V_O2_ max were evaluated pre- and post-exposure to 60-day HDT.

To assess the longitudinal evolution of the variables of interest during the HDT and recovery phases, a paired comparison of these variables between each time point and the baseline was performed in each group. The null hypothesis was that the mean of the population at the given time point was the same as the one in this population at baseline. The comparison was done using a one-sample paired t-test when the differences followed a normal distribution (assessed by Kolmogorov–Smirnov test with Lilliefors correction), or with a Wilcoxon signed-rank test in the opposite case. We use a nonparametric representation of these results because they are more adapted to small sample sizes and give a better picture of the actual distribution of the results.

Then, to get a closer look at the evolution of the variables during exposure to long-duration HDBR, the longitudinal evolution during the HDT phase was further analyzed by fitting separate linear mixed-effects models taking into account the inter-subject and inter-group differences. The fixed effects were the group (CTRL or JUMP), time taken as the day of HDT (baseline records were considered as time = 0), and the interaction between time and group. The random effects were the intercepts for the subjects and the by-subject slopes for the effect of time. Visual inspection of residual plots was performed to detect any obvious deviation from homoscedasticity or normality. p-values were obtained using the Satterthwaite approximations for degrees of freedom, since they lead to acceptable type 1 error rates, even for small sample sizes^33^. In particular, the effect of time and the effect of the countermeasure as the interaction group*time were evaluated. When possible, the variables that could not be analyzed using linear mixed effects models were tested by repeated measures analysis of variance (RM ANOVA).

Some results related to longitudinal evolution, as well as group and group*time effects for SV, V_O2_ max, and plasma volume that have been already reported elsewhere^23,30,31^ are not presented in the tables and figures, but described in the text with appropriate references.

Per-subject slopes were computed using least-square linear regressions of the metrics of interest versus time taken as the day from the beginning of HDT (baseline records were considered as time = 0). Then, correlations between the evolution of PCM and other metrics were performed using these per-subject slopes. Results are expressed as Pearson correlation coefficient R and p-values. One JUMP subject appearing as a clear outlier for SV was removed from the analyses related to this parameter.

All statistical analyses were performed using Matlab (R2017a, MathWorks) and setting two-tailed alpha to reject the null hypothesis at 0.05. Summary data are expressed as median and first and third quartile [Q1; Q3], unless otherwise stated. Comparison of different time points is also reported based on the median value, unless otherwise stated.

## Results

Demographic parameters and baseline measurements of the different metrics of interest are presented in Table 1 for the participants of each group. No intergroup differences were found for most of the metrics, including the demographic parameters, orthostatic tolerance, plasma volume, stroke volume, and almost all the PCM metrics. However, the CTRL and JUMP groups were already different at baseline for ${iK}_{Lin}^{BCG}$ CC (*p* = 0.02) and V_O2_ max (*p* = 0.01).

### Effects of HDBR and recovery

**Figure 3:**
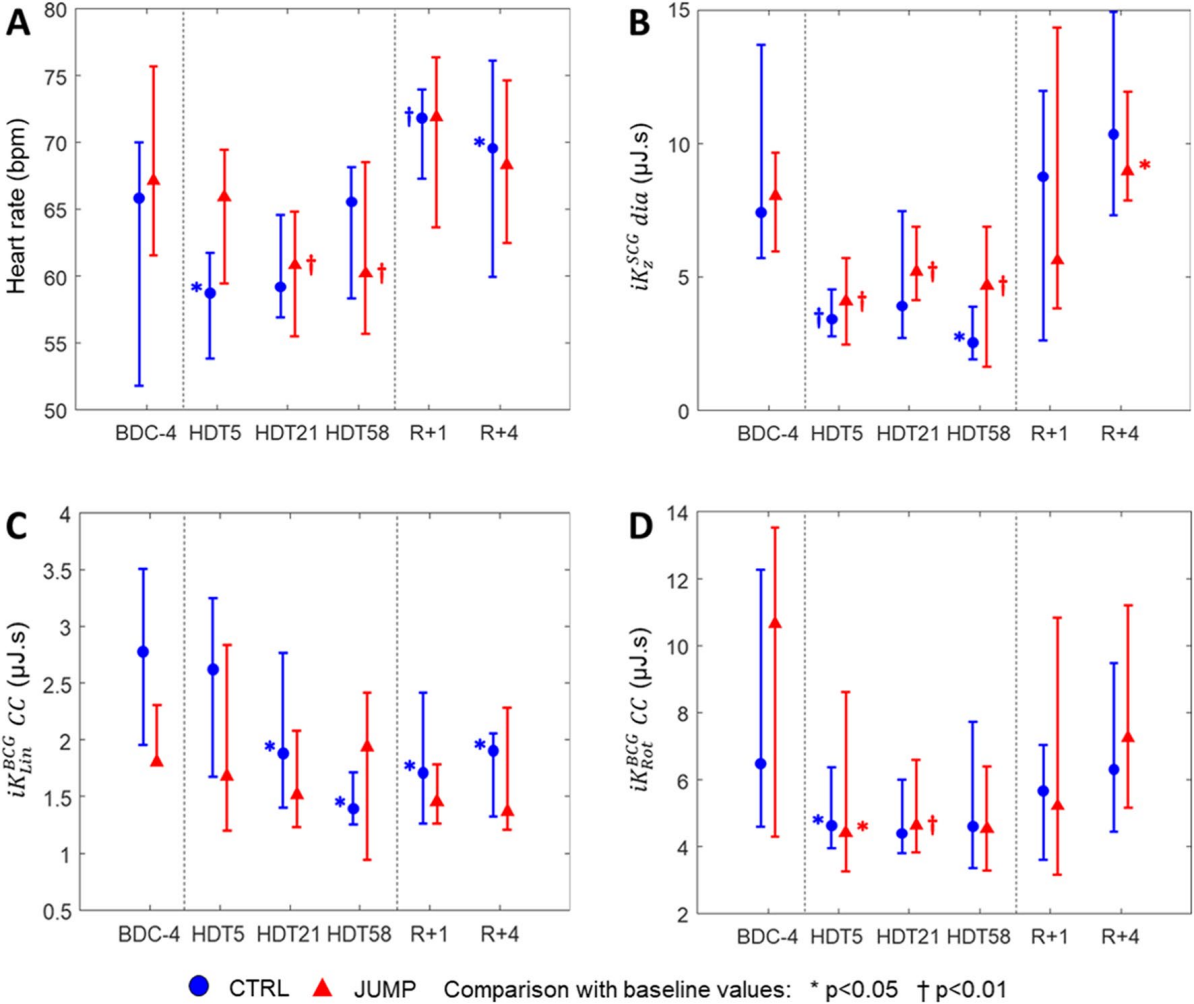
Longitudinal evolution of portable cardiac monitoring metrics along the ESA-RSL study: (**A**) Heart rate (bpm); (**B**) ${iK}_{z}^{SCG}$ during diastole (µJ.s); (**C**) ${iK}_{Lin}^{BCG}$ during a complete cardiac cycle (µJ.s); (**D)** ${iK}_{Rot}^{BCG}$ during a complete cardiac cycle (µJ.s). Results are presented as median [Q1; Q3].

Table 2 presents the time evolution of the chosen metrics in both the CTRL and the JUMP groups, computed based on the PCM device, as well as the results of the orthostatic tolerance tests performed pre- and post-exposure to the 60-day HDT bed rest. Some of these results are also displayed on Fig. 3. The results for the whole cohort combined are presented as a supplementary material.

**Table 2:** Longitudinal evolution of portable cardiac monitoring metrics and orthostatic tolerance along the ESA-RSL study. Results are presented as median [Q1; Q3] for the CTRL and JUMP group. *CC* cardiac cycle, *sys* systole, *dia* diastole. Paired comparison of the metrics with their baseline value: *p < 0.05, ^†^*p* < 0.01.

Metrics		Group	BDC	HDT5	HDT21	HDT58	R + 0/1	R + 4
Heart rate (bpm)		CTRL	66 [52; 70]	59 [54; 62]*	59 [57; 65]	66 [58; 68]	72 [67; 74]^†^	70 [60; 76]*
		JUMP	67 [62; 76]	66 [60; 70]	61 [56; 65]^†^	60 [56; 69]^†^	72 [64; 76]	68 [63; 75]
${{{i}}{{K}}}_{{{L}}{{i}}{{n}}}^{{{B}}{{C}}{{G}}}$(µJ s)	*CC*	CTRL	2.8 [2.0; 3.5]	2.6 [1.7; 3.2]	1.9 [1.4; 2.8]*	1.4 [1.3; 1.7]*	1.7 [1.3; 2.4]*	1.9 [1.3; 2.1]^†^
		JUMP	1.8 [1.8; 2.3]	1.7 [1.2; 2.8]^†^	1.5 [1.2; 2.1]	1.9 [0.9; 2.4]	1.5 [1.3; 1.8]	1.4 [1.2; 2.3]
	*sys*	CTRL	1.6 [1.2; 2.0]	1.5 [1.2; 2.0]	1.3 [0.7; 1.5]	1.0 [0.7; 1.2]*	1.0 [0.7; 1.2]	1.1 [0.8; 1.5]*
		JUMP	1.2 [1.0; 1.3]	0.9 [0.7; 1.6]	1.1 [0.9; 1.4]	1.3 [0.6; 1.6]	0.9 [0.8; 1.2]	0.9 [0.8; 1.1]
	*dia*	CTRL	1.1 [0.7; 1.7]	0.7 [0.5; 1.3]	0.7 [0.3; 1.0]*	0.4 [0.3; 0.7]	0.6 [0.4; 0.9]*	0.6 [0.4; 1.2]^†^
		JUMP	0.7 [0.5; 1.1]	0.7 [0.4; 1.0]	0.5 [0.3; 0.7]*	0.6 [0.3; 0.9]	0.4 [0.4; 0.8]	0.5 [0.3; 1.2]
${{{i}}{{K}}}_{{{R}}{{o}}{{t}}}^{{{B}}{{C}}{{G}}}$(µJ s)	*CC*	CTRL	6.5 [4.6; 12.3]	4.6 [4.0; 6.4]*	4.4 [3.8; 6.0]	4.6 [3.4; 7.7]	5.7 [3.6; 7.0]	6.3 [4.4; 9.5]
		JUMP	10.7 [4.3; 13.5]	4.4 [3.3; 8.6]*	4.6 [3.8; 6.6]^†^	4.5 [3.3; 6.4]	5.2 [3.2; 10.8]	7.3 [5.2; 11.2]
	*sys*	CTRL	4.7 [3.3; 9.0]	3.4 [2.6; 4.4]*	2.9 [2.3; 4.1]	3.0 [2.3; 5.1]	3.6 [1.9; 4.3]	3.7 [2.8; 5.3]
		JUMP	6.5 [3.2; 11.2]	3.1 [2.2; 6.0]*	3.2 [2.6; 4.4]^†^	3.6 [2.0; 5.3]	3.9 [2.2; 6.0]	5.1 [3.2; 8.5]
	*dia*	CTRL	2.0 [1.5; 2.3]	1.4 [1.0; 2.0]	1.5 [1.1; 1.9]	1.2 [0.6; 2.2]	2.1 [1.3; 2.9]	2.3 [1.6; 3.7]
		JUMP	1.9 [1.0; 2.9]	1.3 [0.9; 1.7]	1.4 [1.0; 2.0]	1.0 [0.9; 1.8]	1.4 [0.8; 3.8]	2.1 [1.7; 3.1]
${{{i}}{{K}}}_{{{z}}\boldsymbol{ }}^{{{S}}{{C}}{{G}}}$(µJ s)	*CC*	CTRL	22.8 [11.4; 37.7]	10.2 [7.3; 11.2]^†^	15.6 [12.9; 34.0]	12.0 [8.4; 15.6]*	18.2 [8.2; 30.4]	19.7 [18.8; 25.3]
		JUMP	23.0 [15.4; 28.2]	11.2 [7.3; 15.8]^†^	17.2 [12.3; 23.8]	13.5 [6.6; 23.6]	20.3 [11.8; 54.6]	25.1 [20.3; 36.4]
	*sys*	CTRL	15.2 [7.3; 22.8]	5.5 [4.5; 7.9]*	12.0 [10.2; 25.4]	8.5 [7.2; 12.7]	9.9 [5.9; 18.5]	10.7 [8.5; 11.6]
		JUMP	11.9 [8.2; 21.7]	7.2 [4.7; 9.2]*	12.5 [8.1; 17.3]	8.9 [4.9; 18.9]	16.5 [7.4; 37.4]	16.5 [11.3; 26.8]
	*dia*	CTRL	7.4 [5.7; 13.7]	3.4 [2.8; 4.6]^†^	3.9 [2.7; 7.5]	2.5 [1.9; 3.9]*	8.8 [2.6; 12.0]	10.4 [7.3; 14.9]
		JUMP	8.0 [6.0; 9.7]	4.1 [2.5; 5.7]^†^	5.2 [4.1; 6.9]^†^	4.7 [1.6; 6.9]^†^	5.6 [3.8; 14.4]	9.0 [7.9; 11.9]*
Orthostatic tolerance (min)		CTRL	22.4 [20.3; 24.7]	–	–	–	6.4 [2.7; 15.9]^†^	–
		JUMP	23.3 [22.2; 23.9]	–	–	–	12.6 [6.2; 16.5]^†^	–

Compared to BDC, the orthostatic tolerance was greatly decreased after 60 days of HDBR in both groups: − 71% for CTRL and − 46% for JUMP (both *p* < 0.01). Caiani et al. already described a similar evolution for SV in the participants of this study (− 22% in CTRL and − 12% in JUMP)^31^ and so did Kramer et al. for the plasma volume (− 13% in both CTRL and JUMP)^23^. However, the mean V_O2_ max has been reported to be significantly reduced only in the CTRL group (− 29%)^30^.

In the CTRL group, a transient decrease in heart rate was noticeable at HDT5 when compared to BDC (− 11%, *p* < 0.05), but it returned towards baseline values during the rest of the HDT phase and increased during recovery (+ 9%, *p* < 0.01 and + 6%, *p* < 0.05 at R + 1 and R + 4, respectively). The same trend was not observed in the JUMP group, for which heart rate only decreased at HDT21 (− 9%, *p* < 0.01) and HDT58 (− 10%, *p* < 0.01), compared to baseline.

${iK}_{Lin}^{BCG} CC$ was slightly lower than baseline at HDT5 in the JUMP group (− 6%, *p* < 0.01), while such a decrease was observed later in the CTRL group: at HDT21 and HDT58 (− 32%, *p* < 0.05 and − 50%, *p* < 0.05, respectively). Following early reambulation, ${iK}_{Lin}^{BCG} CC$ in the CTRL group was still lower than BDC at R + 4 (− 32%, *p* < 0.01).

The energy of the rotational movements, as assessed by ${iK}_{Rot}^{BCG} CC$, was decreased as early as at HDT5 (− 29% in CTRL and − 59% in JUMP, both *p* < 0.05). This change was likely caused by the systolic component, for which we observed similar changes, while the diastolic component remained unchanged during the entire study. All the rotational BCG metrics were back to their baseline values as early as at R + 1.

Regarding the precordial movements, ${iK}_{z}^{SCG}$ measured on the whole cardiac cycle decreased during the HDT phase, in particular at HDT5 (− 55% in CTRL and − 51% in JUMP, both *p* < 0.01). The evolution of ${iK}_{z}^{SCG} CC$ was not monotonous with time and this phenomenon seemed to be mainly associated with the systolic contribution. As regards the diastolic component of ${iK}_{z}^{SCG}$ (see Fig. 3B), it remained relatively stable during HDT after an initial decrease at HDT5 (− 54% in CTRL and − 49%, both *p* < 0.01). For all cardiac phases, ${iK}_{z}^{SCG}$ returned to its baseline value as early as at R + 1, but with very high inter-subject variability. An overshoot was observed at R + 4, for ${iK}_{z}^{SCG} dia$ in JUMP (+ 13%, *p* < 0.05).

### Effects of countermeasure during and after HDBR

As suggested by the results presented in Table 2 and Fig. 3, some metrics did not follow a monotonous evolution during HDT. Table 3 presents the results of the linear mixed-effects model statistical analysis for the metrics that passed the visual inspection of the residual plots.

**Table 3:** Results of the linear mixed-effects model statistical analysis for heart rate, ${\mathrm{iK}}_{\mathrm{Lin}}^{\mathrm{BCG}}$ (during the whole cardiac cycle, systole, and diastole), ${\mathrm{iK}}_{\mathrm{z}}^{\mathrm{SCG}}$ (during diastole), orthostatic tolerance, and stroke volume. The fixed effects are the group (CTRL or JUMP), HDT time (baseline records being considered as time = 0), and the interaction between HDT time and group. p-values displayed in the table are obtained using the Satterthwaite approximations for degrees of freedom.

Metrics		Effect of time	Effect of time* group
Heart rate		*p* = 0.005	*p* < 0.001
${{{i}}{{K}}}_{{{L}}{{i}}{{n}}}^{{{B}}{{C}}{{G}}}$	*CC*	*p* < 0.001	*p* = 0.013
	*sys*	*p* < 0.001	*p* = 0.001
${{{i}}{{K}}}_{{{z}}}^{{{S}}{{C}}{{G}}}$	*dia*	*p* = 0.017	*p* = 0.400
	*dia*	*p* < 0.004	*p* = 0.167
Orthostatic tolerance		*p* < 0.001	*p* = 0.205
Stroke volume		*p* < 0.001	*p* = 0.005

In agreement with previous results, HDT exposure had a statistically significant effect on all the variables tested by linear mixed-effects statistical analysis (see Table 3).

Kramer and colleagues have already shown that there was a significant time*group effect for V_O2_ max during this study^30^, marking the efficacy of the countermeasure in the JUMP group, but that such an effect could not be found for plasma volume^23^.

The interaction between group and HDT time had a significant effect on SV (*p* = 0.005) and on heart rate (*p* < 0.001), in agreement with the different trends for CTRL and JUMP observed in Table 2 and Fig. 3A.

${iK}_{Lin}^{BCG}\boldsymbol{ }CC$ was also affected by the interaction between group and HDT time (*p* = 0.013), mainly via its systolic contribution (*p* = 0.001). As reported in Table 2 and Fig. 3C, ${iK}_{Lin}^{BCG}\boldsymbol{ }CC$ was indeed relatively stable all along the HDT phase in the JUMP group, while it decreased progressively in the CTRL group.

For the remaining metrics tested by linear mixed-effects model analysis, the countermeasure did not show any significant effect. This is for instance the case of orthostatic tolerance, ${iK}_{Lin}^{BCG} dia$, and ${iK}_{z}^{SCG} dia$.

The metrics that did not follow a linear evolution with regard to HDT time also exposed no significant effect of the interaction between group and HDT time, as assessed by RM ANOVA. One of these metrics is ${iK}_{Rot}^{BCG} CC$, displayed on Fig. 3D.

### Correlations between the trends of PCM and other parameters

In such a context and given the low number of subjects in this study, the trends of the evolution of the different parameters with time may be more informative than the simple comparison of the repeated measurements. It is this trend between baseline and all the measurement points of the HDT phase that has been used to correlate PCM metrics with other markers of the cardiovascular condition.

**Figure 4:**
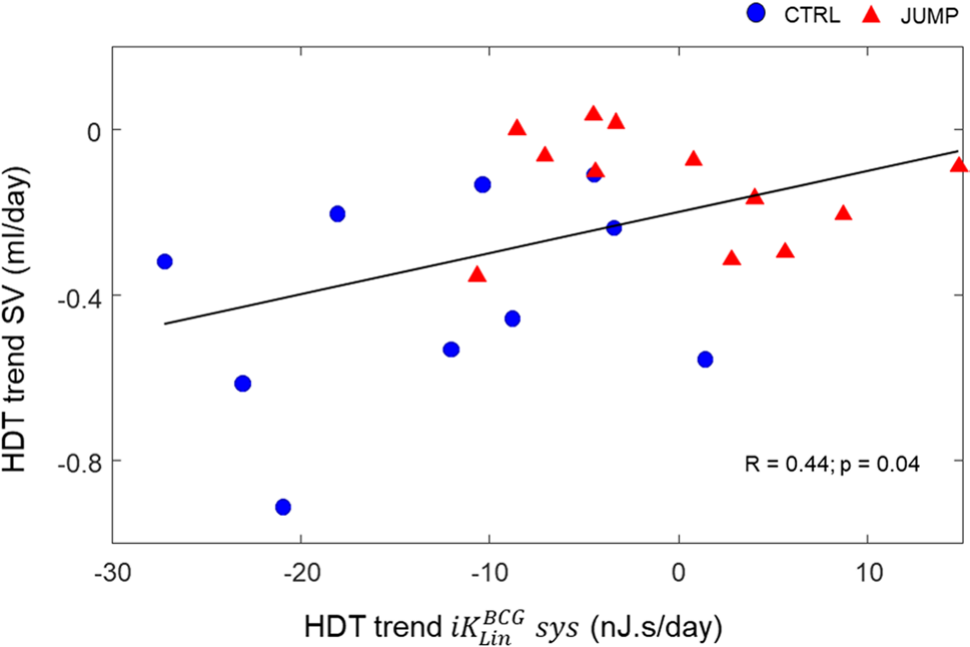
Scatter plot of the per-subject HDT trends for stroke volume (SV) and ${iK}_{Lin}^{BCG}\boldsymbol{ }sys$ in the CTRL and JUMP groups. Baseline records are considered as time = 0. Correlation result is expressed as Pearson correlation coefficient R and p-value.

The results are presented in Table 4 and highlight a correlation between the evolution of SV and the ones of heart rate (R =  − 0.72, *p* < 0.01), ${iK}_{Lin}^{BCG}\boldsymbol{ }sys$ (R = 0.44, *p* < 0.05), and ${iK}_{z}^{SCG} dia$ (R = 0.46, *p* < 0.05). The example of the distribution of trends in SV versus ${iK}_{Lin}^{BCG}\boldsymbol{ }sys$ is displayed on Fig. 4 and shows a clear distinction between CTRL participants (negative HDT trends for SV and ${iK}_{Lin}^{BCG}\boldsymbol{ }sys$) and JUMP participants (trends for SV and ${iK}_{Lin}^{BCG}\boldsymbol{ }sys$ both closer to zero).

**Table 4:** Results of the correlation analysis between HDT trend of the parameters of the portable cardiac monitoring system and HDT trend of other markers of the cardiovascular condition. *p < 0.05, ^†^*p* < 0.01.

	Heart rate	Stroke volume	V_O2_ max	Plasma volume	Orthostatic tolerance
Heart rate		R = − 0.72^†^	R = − 0.52*	R = − 0.11	R = − 0.36
${iK}_{Lin}^{BCG} sys$	R = − 0.58^†^	R = 0.44*	R = 0.47*	R = − 0.04	R = 0.09
${iK}_{Lin}^{BCG} dia$	R = − 0.18	R = − 0.17	R = − 0.18	R = − 0.09	R = − 0.14
${iK}_{z}^{SCG} dia$	R = − 0.23	R = 0.46*	R = 0.17	R = − 0.25	R = − 0.05

A correlation was also found between the trends of ${iK}_{Lin}^{BCG}\boldsymbol{ }sys$ and heart rate (R =  − 0.58, *p* < 0.01), ${iK}_{Lin}^{BCG}\boldsymbol{ }sys$ and V_O2_ max ( R = 0.47, *p* < 0.05), as well as heart rate and V_O2_ max (R =  − 0.52, *p* < 0.05).

The evolutions of PCM metrics were not correlated to the ones of plasma volume and orthostatic tolerance.

## Discussion

The evolution of the cardiovascular status in 22 participants was longitudinally assessed during a HDBR study with a HDT duration of 60 days by using novel metrics extracted through a PCM system that combined ECG, SCG, and BCG measurements. Several PCM metrics have been found to be affected by long-duration exposure to HDT and to expose changes that correlate with the simultaneous decrements in SV and V_O2_ max. Since a positive effect of the countermeasure was shown for SV and V_O2_ max, this effect was then also visible on some PCM metrics and in particular ${iK}_{Lin}^{BCG}\boldsymbol{ }sys$.

### Cardiovascular effects of long-term HDBR

In the CTRL group, the initial decrease in supine heart rate, followed by a return to baseline values later during the HDT phase, and a marked increase in the first days of reambulation is in agreement with other studies published on the same participants^23,34^, but also with other HDBR studies^2,35^. The analysis of heart rate variability performed on the participants of this study by Maggioni and colleagues suggests that the trend observed for heart rate in CTRL could be caused by a reduction of vagal modulation and an increase in the sympathovagal balance^34^. Another element to take into account to interpret this trend for heart rate is given by the progressive decrease in SV assessed by impedance cardiography^34^ and CMR^31^ in these participants and the need to keep a reasonable cardiac output, as underlined by the strong correlation between the trends of heart rate and SV in Table 4.

In the present study, such a non-linear trend with regard to HDT time is also seen in both groups on ${iK}_{z}^{SCG}$ *CC*, as well as its systolic component. Previous studies have shown that ${iK}_{z}^{SCG} CC$ and SV assessed by echocardiography were positively correlated in an increased contractility setting based on infusion of increasing doses of Dobutamine^29^. Other studies have also found very good correlations between different SCG-based metrics and SV both without intervention^36^ or during exposure to lower-body negative pressure^37^. In this HDBR study, the evolution of ${iK}_{z}^{SCG}$ *CC* cannot solely be due to changes in SV. Indeed, as already described, SV followed a monotonous decreasing trend with regard to HDT time. The decrease in plasma volume during HDBR has constantly been reported to occur relatively quickly after the beginning of the HDT phase and should have already stabilized by HDT5^38^. These early changes certainly had a negative effect on preload and atrial pressure, which may explain the initial drop in ${iK}_{z}^{SCG}$ *CC*, but not the following evolution, suggesting that other factors must explain the observed mid- and long-term effects. These could include adaptation of the central autonomous nervous system and its effect on loading and strain, as well as apparent decrease in LV mass, which has been shown to be very progressive during HDBR^3^ and might be due to dehydration rather than cardiac apoptosis^39,40^.

The quick and stable drop in the diastolic ${iK}_{z}^{SCG}$ during the HDT phase, followed by a fast recovery on return to normal ambulation in both groups, as seen in Fig. 3B, may be indirectly linked to changes in plasma volume, even though no correlation was found. Indeed, changes in plasma volume also occur very quickly and similarly in both groups^23^, and may affect filling of the ventricles, as underlined by the reduction observed in these participants in the mitral inflow parameters and in particular the early filling peak flow rate^31^. This interpretation is also supported by the quick decrease of ${iK}_{Rot}^{BCG} CC$ at the beginning of the HDT phase (see Fig. 3D). Indeed, the activity recorded by the rotational channels of BCG could be linked to the twisting and untwisting movements of the heart, which are also influenced by the pressure in the left ventricle^41^. However, these explanations remain very speculative, since long-term HDBR may also have an impact on the exact position and orientation of the heart, as well as changes in the mechanical coupling between the heart and the chest wall. Measuring precordial movements in six dimensions (three linear and three angular movements), rather than only linear accelerations on the z axis, could help alleviate this issue.

On Fig. 3C, a decreasing trend is observed all along the HDT phase for ${iK}_{Lin}^{BCG} CC$ in the CTRL group. This observation is to put in context with a previous study that we conducted in an increased contractility setting^29^, where we found that the most significant correlation of our SCG/BCG metrics with SV was given by ${iK}_{Lin}^{BCG} CC$ (R = 0.73). In this HDBR study, we have also found that the progressive decrease in SV and ${iK}_{Lin}^{BCG} sys$ were closely related (see Table 4 and Fig. 4). ${iK}_{Lin}^{BCG} sys$ is then apparently also able to easily reflect changes in SV in the case of decreased contractility, without requiring CMR or echocardiography systems. At the end of the HDT phase, this decrease of ${iK}_{Lin}^{BCG} sys$ – and thus SV – while resting heart rate was unchanged, may also partly explain why Kramer et and colleagues found that the cardiorespiratory fitness was decreased in the CTRL group^30^. This assumption is confirmed by the positive correlation (R = 0.47, *p* < 0.05) that was found between the HDT trends for ${iK}_{Lin}^{BCG} sys$ and V_O2_ max.

The decrease in orthostatic tolerance observed in the CTRL group was very much expected and reproduces what has been observed during previous HDBR studies and spaceflights. The absence of correlation between the decrement of orthostatic tolerance and the evolution of the PCM metrics supports the fact that post-HDBR orthostatic intolerance is multifactor and the result of a complex interplay between hypovolemia, autonomic nervous system regulation modifications, increase in venous distensibility, and hormonal and metabolic changes^38^.

### Efficacy of the applied countermeasure

The different evolution of heart rate in the two groups (*p* < 0.001), with a decrease in the JUMP group (− 9% and − 10% at HDT21 and HDT58, respectively, *p* < 0.01), highlights a positive effect of the chosen countermeasure. The initial decrease of heart rate seen in the whole cohort is consistent with spaceflight data on short-duration flights^42^, but the subsequent increase observed in the CTRL group is not seen on astronauts during long-duration flights^43^, probably because astronauts have daily physical exercise regimes during their stay in weightlessness. From this perspective, the evolution of heart rate in the JUMP group is closer to what is observed on the astronauts than the one in the CTRL group.

As reported by Caiani and colleagues^31^ and further studied here, SV measured by CMR in the participants of this study decreased progressively during the HDT period, with a steeper slope in CTRL than in JUMP (− 28% vs. − 9.2% at HDT58, respectively). Similarly, a decreasing trend was observed all along the HDT phase for ${iK}_{Lin}^{BCG} CC$ and ${iK}_{Lin}^{BCG} sys$ in the CTRL group, while they remained unchanged in the JUMP group (see Table 2 and Fig. 3C). On Fig. 4, a scatter plot of the HDT trends for SV versus ${iK}_{Lin}^{BCG} sys$ allows a clear distinction between the two groups. However, interestingly, the slight and progressive decrease measured in SV for the JUMP participants during HDT is not reflected by a similar trend in ${iK}_{Lin}^{BCG} CC$ or ${iK}_{Lin}^{BCG} sys$. This may be partly due to differences between the PCM protocol and the one of CMR, as well as specific testing schedule of these protocols. Indeed, for some—but not all—of the measurements taking place during the HDT phase, the participants had to change from HDT to horizontal position to perform a one-hour brain MRI that was followed by a one-hour CMR, still in horizontal position, before coming back to HDT position to perform the PCM protocol. Therefore, venous return and pre-load were transiently decreased during the MRI protocols, before being increased again just afterwards for the PCM protocol. In addition, CMR protocols are conducted in a noisy and stressful environment, often requiring the participants to hold their breath during imaging. Even though the flow protocol used to measure SV in this study did not require breath holds, other sequences conducted before this flow measurement had such requirements, which may have impacted the actual values of SV in the participants. This point is also supported by the fact that some of our previous studies showed that breath holds as short as 10 s led to large increases in ${iK}_{Lin}^{BCG} CC$ in healthy subjects^44^. This research protocol was not designed for studying close correlation between the two methods, but rather to evaluate their overall agreement in evaluating cardiac adaptation, which was successfully done.

After 60 days of HDBR, Kramer and colleagues^30^ found a significant decrease of V_O2_ max in the CTRL group, whereas it was stable in the JUMP group. Even though baseline values were different between these two groups, statistical analysis showed that the tested countermeasure may have had a positive effect regarding maximal aerobic capacity. The difference between these two groups cannot be explained by intergroup differences regarding blood volume, because they changed similarly in both groups^23^. Among the possible explanations of this behavior is the different evolution of resting heart rate and SV in the two groups. By keeping a relatively high resting SV and low resting heart rate, a well-known effect of physical exercise^45^, the JUMP participants may have maintained more cardiorespiratory fitness than the CTRL participants. Alternatively, for V_O2_ max, SV, and ${iK}_{Lin}^{BCG} CC$, the different evolutions observed between groups could also be at least partially due to an effect of regression to the mean, because the CTRL group was globally fitter than the JUMP group at baseline.

The countermeasure of the current study was designed to maintain muscle and bone mass during HDBR, for which it was very effective^23^, and though it seemed to have some beneficial effects on cardiac function and cardiorespiratory fitness, it had little to no positive effect on orthostatic tolerance. Maggioni and colleagues^34^ found a reduced cardiac autonomic deconditioning in JUMP, with no apparent alterations during HDT, but studies have shown that post-HDBR orthostatic intolerance was not directly linked to the degree of autonomic cardiac adaptation^35^. Previous experimentation of high intensity resistive and rowing exercise during 70 days of HDBR did also not prevent a loss in orthostatic tolerance^46^. Indeed, successful countermeasures against post-HDBR orthostatic intolerance combined both exercise and volume loading^47,48^. The positive effect of the type of training chosen in this study should then maybe be combined with restoration of the plasma volume loss in the last days of exposure to artificial microgravity. This could make it more efficient regarding orthostatic tolerance, in addition to the positive effects already observed on heart rate, resting SV, and cardiorespiratory fitness.

### Lessons learned for the use of SCG and BCG

Figure 3C,D show the differences in the time course of changes in ${iK}_{Lin}^{BCG} CC$ and ${iK}_{Rot}^{BCG} CC$ in the two groups. One can argue that their evolution during this HDBR study are caused by different phenomena. Moreover, the evolution of the different cardiac phases of ${iK}_{Lin}^{BCG}$ is very interesting, even if the BCG signal cannot be absolutely synchronous with the cardiac events defined using the ECG, as opposed to the SCG signal recorded directly on the chest. Indeed, the results of the linear mixed-effects model statistical analysis show no group*time effect for the diastolic phase of ${iK}_{Lin}^{BCG}$ (*p* = 0.4), but a very significant effect for its systolic phase (*p* = 0.001). The differences in the evolution of the metrics of the PCM system not only prove the complementarity of BCG and SCG but also highlight the added value to separate systolic and diastolic phases of the cardiac cycle, as well as linear and rotational movements in the computation of the $iK$ parameters.

After recently proving that these techniques could detect a state of increased contractility^29^, this study gives insight into the possibility to monitor over time a deconditioning state of the cardiovascular system.

### Limitations

Several possible limitations require consideration and the first one is linked to the schedule of the measurements. Indeed, HDBR studies involve many scientists, who must fit their experiments in a very tight schedule with limited access to some facilities. As a consequence, during this study, the PCM measurements were occasionally conducted after a brain and/or a heart MRI in supine horizontal position. When it was the case, such a long exposure to an MRI environment in horizontal position may have affected the hemodynamic status of the subjects, especially during the HDT phase. For future studies, we recommend reversing the order of these experiments and rather conducting SCG and BCG assessment prior to the MRI protocols.

The SCG signal is relatively sensitive to the exact position of the sensor, which can lead to inter-and intra-subject differences^49^. Here we always targeted the same place for the SCG sensor: the apex, using anatomical references. Recent studies have also shown that a larger contact area could prevent SCG dependence on sensor placement^50^. These authors reported that for a contact area of 3.5 cm^2^, a 1 cm displacement led to 5% difference on the root-mean-square power amplitude. We can expect an even lower dependence on the exact position for a sensor with a contact area of 8.4 cm^2^, such as the one we used. This point must be carefully considered, since we can expect changes in the exact position and orientation of the heart between horizontal and HDT positions. This limitation could be partially overcome by measuring SCG in three dimensions rather than on only one axis^51^. In addition, recording the rotational movement of the SCG has also already proved to be useful^52^ and could consolidate the measurement of the metrics of interest or bring additional information. Ideally, SCG should include measurements of three-axis linear accelerations and angular velocities.

Like the cardiac cycle itself, SCG and BCG signals are sensitive to breathing and this is the reason why an ICB protocol was used in the present study. Such ICB protocols do not reproduce spontaneous breathing patterns, but these limitations apply to CMR and echocardiography as well.

Cardiac phases of the SCG and BCG signals have been defined using the synchronous ECG signal. However, there is a delay between the Q wave of the ECG and the characteristic systolic complex that can be observed on BCG. Because of this phase offset, it is possible that some of the systolic activity was recorded at the beginning of the diastolic phase defined using the ECG.

Due to some technical problems of the z-axis of linear BCG for some records, we decided to remove it from all our analyses, which may have increased the inter- and intra-subject variability. This was, however, the best compromise in order not to remove otherwise valid records from our data set.

With a mean age of 29 years old, the participants of this study are all relatively young and it is already known that consequences of bed rest are influenced by the age^53^. However, a previous study has found a larger decrease in V_O2_ max for older men than for younger men^53^. According to the observed correlations between BCG metrics and V_O2_ max, it is reasonable to expect that larger decreases would also have been observed in older men for SCG and especially BCG. These effects of (simulated) microgravity on SCG and BCG would then also be detected, validating also the interest of a PCM device such as the one presented for older populations, including astronauts.

Finally, some baseline differences existed between the JUMP and CTRL subjects. This is an inherent problem of studies with a low number of subjects, which is very difficult to solve in the context of complex HDBR studies. In particular, the fact that the CTRL group was globally fitter than the JUMP group may have caused a regression to the mean effect.

Nonetheless, the authors believe that these limitations did not preclude any of the conclusions of this research.

## Conclusion

In the present study, we evaluated the effects of 60 days of HDT on the cardiovascular system and assessed the efficacy of an exercise-based countermeasure consisting of high-intensity jump training. In particular, we investigated the ability of a PCM system based on SCG and BCG to assess cardiovascular deconditioning caused by long-duration HDBR and compared the results to gold standard techniques. This is the first study to our knowledge to evaluate calibrated SCG and multi-dimensional BCG as a marker of the cardiovascular state during HDBR deconditioning.

The main findings of the present investigation are threefold. Firstly, we showed that the cardiovascular adaptations that occurred during HDT were reflected in different metrics of the PCM. In particular, the evolution of ${iK}_{Lin}^{BCG} sys$ followed the changes in SV and V_O2_ max in both groups.

Secondly, the different responses to HDBR in resting heart rate and ${iK}_{Lin}^{BCG}$ between the control and countermeasure groups showed that the PCM system based on SCG and BCG was able to demonstrate the effectiveness of the applied countermeasure on the cardiovascular system during long-duration exposure to HDT. This, coupled to the positive effect it had on cardiorespiratory fitness, shows that short-duration high-intensity jump training may at least partially have counteracted cardiovascular deconditioning.

Thirdly, we have shown the importance to discriminate the different cardiac phases in the analysis of SCG and BCG signals, but also to record these precordial and global movements of the body in parallel and in both linear and rotational dimensions. We recommend that future SCG and BCG systems use sensors with 3-axis accelerometers and 3-axis gyroscopes in order to measure the vibrations induced by cardiovascular activity in their entirety.

## Supplementary information

Supplementary information.
