# Supplementary material for: Cardiovascular adaptation to simulated microgravity and countermeasure efficacy assessed by ballistocardiography and seismocardiography
Source: Sci Rep. 2020 Oct 19;10:17694. doi: 10.1038/s41598-020-74150-5 (PMC7573608; doi:10.1038/s41598-020-74150-5)
Supplement: Supplementary file 1 — Supplementary information. [file 41598_2020_74150_MOESM1_ESM.docx]

## Supplementary material

Cardiovascular adaptation to simulated microgravity and countermeasure efficacy assessed by ballistocardiography and seismocardiography

Jérémy Rabineau*^1,2^, Amin Hossein^1^, Federica Landreani^3^, Benoit Haut^2^, Edwin Mulder^4^, Elena Luchitskaya^5^, Jens Tank^4^, Enrico G. Caiani^3^, Philippe van de Borne^6^, Pierre-François Migeotte^1^

^1^ LPHYS, Université libre de Bruxelles, Brussels, Belgium. ^2^ TIPs, Université libre de Bruxelles, Brussels, Belgium. ^3^ Electronic, Information and Biomedical Engineering Dpt., Politecnico di Milano, Milan, Italy. ^4^ Institute of Aerospace Medicine, German Aerospace Center (DLR), Cologne, Germany. ^5^ IMBP, Russian Academy of Sciences, Moscow, Russian Federation. ^6^ Department of Cardiology, Erasme Hospital, Université libre de Bruxelles, Brussels, Belgium.

Contact for correspondence: [Jeremy.Rabineau@ulb.ac.be](mailto:Jeremy.Rabineau@ulb.ac.be)

Table S1 – Longitudinal evolution of portable cardiac monitoring metrics and orthostatic tolerance along the ESA-RSL study. Results are presented as median [Q1; Q3] for the whole cohort (CTRL and JUMP together). CC: cardiac cycle; sys: systole; dia: diastole. Paired comparison of the metrics with their baseline value: ^*^ p<0.05, ^†^ p<0.01.

| Metrics | | BDC | HDT5 | HDT21 | HDT58 | R+0/1 | R+4 |
| --- | --- | --- | --- | --- | --- | --- | --- |
| Heart rate (bpm) | | 66 [61; 72] | 61 [58; 67] **^†^** | 60 [57; 65] **^†^** | 64 [57; 68] | 72 [65; 76] **^†^** | 70 [62; 75] **^†^** |
|  | *CC* | 2.0 [1.8; 2.7] | 2.1 [1.6; 3.0] | 1.6 [1.3; 2.2] **^†^** | 1.7 [1.1; 2.2] **^†^** | 1.5 [1.3; 2.1] **^†^** | 1.6 [1.3; 2.1] **^†^** |
| $\boldsymbol{iK}_{\boldsymbol{Lin}}^{\boldsymbol{BCG}}$*(µJ.s)* | *sys* | 1.2 [1.1; 1.6] | 1.3 [0.9; 1.9] | 1.1 [0.8; 1.5] | 1.1 [0.7; 1.4] **^*^** | 0.9 [0.8; 1.2] **^*^** | 1.0 [0.8; 1.3] **^*^** |
|  | *dia* | 0.8 [0.6; 1.5] | 0.7 [0.5; 1.2] | 0.5 [0.3; 0.9] **^†^** | 0.5 [0.3; 0.9] **^†^** | 0.5 [0.4; 0.8] **^†^** | 0.6 [0.4; 1.2] **^*^** |
|  | *CC* | 7.7 [4.6; 12.3] | 4.4 [3.4; 6.4] **^†^** | 4.5 [3.8; 6.0] **^†^** | 4.6 [3.4; 6.7] **^*^** | 5.2 [3.3; 9.6] **^*^** | 7.3 [4.4; 10.0] |
| $\boldsymbol{iK}_{\boldsymbol{Rot}}^{\boldsymbol{BCG}}$*(µJ.s)* | *sys* | 5.6 [3.3; 10.2] | 3.1 [2.3; 4.4] **^†^** | 3.0 [2.5; 4.1] **^†^** | 3.1 [2.1; 5.1] **^*^** | 3.6 [2.1; 5.3] **^*^** | 4.4 [2.8; 7.0] |
|  | *dia* | 2.0 [1.2; 2.6] | 1.4 [0.9; 1.8] | 1.4 [1.0; 1.9] | 1.0 [0.8; 1.9] | 1.8 [1.1; 3.3] | 2.2 [1.6; 3.5] |
|  | *CC* | 23.0 [13.9; 33.6] | 10.5 [7.3; 15.0] **^†^** | 16.6 [12.9; 25.3] | 12.8 [7.9; 21.1] **^*^** | 19.3 [10.5; 46.2] | 22.9 [18.8; 36.3] |
| $\boldsymbol{iK}_{\boldsymbol{z}}^{\boldsymbol{SCG}}$ *(µJ.s)* | *sys* | 11.9 [7.6; 21.9] | 6.4 [ 4.6; 9.0] **^†^** | 12.4 [8.2; 21.9] | 8.5 [6.0; 15.2] | 12.2 [6.7; 24.5] | 12.0 [10.1; 20.5] |
|  | *dia* | 8.0 [5.8; 10.7] | 3.8 [2.5; 5.5] **^†^** | 4.7 [3.4; 7.2] **^†^** | 2.7 [1.7; 5.4] **^†^** | 7.9 [3.4; 13.6] | 9.1 [7.5; 12.8] **^*^** |
| Orthostatic tolerance (min) | | 22.9 [22.0; 24.2] | / | / | / | 9.6 [3.7; 16.3] **^†^** | / |
